# The value of a mobile educative Application additional to Standard counselling on aspirin Adherence in Pregnancy: the ASAP study, a randomised controlled trial

**DOI:** 10.1016/j.pecinn.2024.100268

**Published:** 2024-02-18

**Authors:** Jeske M. bij de Weg, Marjon A. de Boer, Cynthia Meijer, Noëlle Lugtenburg, Marijke Melles, Johanna I.P. de Vries, Christianne J.M. de Groot

**Affiliations:** a Amsterdam UMC location Vrije Universiteit Amsterdam, Department of Obstetrics and Gynaecology, Amsterdam, Netherlands; b Amsterdam Reproduction and Development Research Institute, Amsterdam, Netherlands; c Delft University of Technology, Faculty of Industrial Design Engineering, Department of Human-Centered Design, Delft, Netherlands

**Keywords:** Adherence, Aspirin, Counselling, Education, Educative application, Pregnancy

## Abstract

**Objective:**

To assess the added value of a novel, mobile educative application to standard counselling on aspirin adherence during pregnancy versus standard counselling alone.

**Methods:**

Participants were randomly assigned for additional use of a mobile educative application or standard counselling alone. Main outcome measures were adherence to aspirin measured by two validated questionnaires: Simplified Medication Adherence Questionnaire (SMAQ), Believes and Behaviour Questionnaire (BBQ), and patients reported tablet intake >90%.

**Results:**

A total of 174 women with an indication for aspirin during pregnancy were included. The questionnaires were filled in by 126 out of the 174 participants (72.4%). Similar results were found in the app group and the standard counselling groups for both validated questionnaires. Tablet intake >90% was seen in 88.7% and 87.5% (*p* = 0.834) of the app group and standard counselling group respectively. Subgroup analyses demonstrated a negative effect of BMI and a positive effect of educational level on adherence.

**Conclusions:**

Our study revealed no added effect of a novel, mobile educative application to standard counselling on aspirin adherence during pregnancy. Tablet intake was equally high in both groups probably explained by our high educated population.

**Innovation:**

Future studies should focus on tailored counselling on medication to pregnant women's needs including medication reminders, addressing concerns, adequate health literacy and side effects, offering rewards to further stimulate aspirin adherence in pregnancy with optimal outcome for mother and their neonate.

## Highlights

• The risk-reducing effect of aspirin is suggested to be related to therapy adherence.
• We hypothesized that an educative application may improve adherence to aspirin during pregnancy.
• No added effect of an educative application to standard counselling on aspirin adherence was found.
• Tablet intake >90% was 88.7% in the app group and 87.5% in the standard counselling group.

## 1 Introduction

Preeclampsia is a common complication of pregnancy contributing to maternal and perinatal morbidity and mortality [[1], [2], [3], [4]]. Unfortunately, no curative treatment for preeclampsia exists, but low-dose aspirin can reduce the risk of developing preeclampsia in women at risk and is nowadays world-wide used [1,5,6]. The exact working mechanism of aspirin in reducing this risk is unknown, but it is probably explained by its antiplatelet and anti-inflammatory activity, resulting in less abnormal placentation [1].

The risk-reducing effect of aspirin is suggested to be related to therapy adherence. Secondary analyses of the ASPRE trial, in which the preventive effect of aspirin on preterm preeclampsia was investigated in a placebo-controlled setting, showed that in case of aspirin adherence rates ≥90% a stronger risk-reducing effect was seen in comparison to rates <90% (OR 0.24; 95% CI 0.09–0.65 versus OR 0.59; 95% CI 0.23–1.53) [7,8]. Mone et al. concluded that in case of confirmed adherence, aspirin non-responsiveness did not exist [9]. Non-adherence rates of aspirin during pregnancy of up to 46% were reported [10]. These insights about aspirin adherence in pregnancy, emphasize the need for improvement of the adherence on aspirin in the prevention of pre-eclampsia, especially since no curative treatment exists.

Several factors influence aspirin adherence during pregnancy: pill burden and non-intentional omission have a negative influence, and good communication and a good relationship with health care providers have a positive effect [11]. In the latter article, however, no clear definition of good patient-health care provider relation is provided, since it was the interpretation of the women themselves. Citations of the interviews revealed factors as ‘trust’ and ‘confidence’. Previous studies into the relation between the use of a mobile application and medication adherence showed positive effects on adherence, however, the effect on pill burden, non-intentional omission or knowledge was not tested [[12], [13], [14]]. Also improving knowledge with oral and written information increased aspirin adherence during pregnancy [15]. These data suggest investing in education might help improving adherence. A novel device such as an application (mHealth) could be used to respond to the current way of collecting information and health literacy. The motivation to use a mHealth application is based on the results of a systematic review on the effect of mobile application on medication adherence [14]. In seven out of eleven studies, the mobile application increased adherence, and in five studies the significant improvement ranged from 7% to 40% [14]. The mobile applications were easy to use with average satisfaction scores of 8.1 out of 10 [14].

Therefore, in this RCT, we assessed the added value of the use of an educative application on aspirin adherence during pregnancy to standard counselling versus standard counselling alone. We hypothesized that the use of an educative application might improve adherence to aspirin during pregnancy mediated by enhanced knowledge.

## 2 Methods

### 2.1 Study population

Inclusion criteria were an indication for using aspirin during pregnancy according to the Dutch national guideline [16], which is based on NICE Guideline [17], and maternal age of ≥18 years. Exclusion criterion was the inability to read the Dutch language, because the mobile application was only available in Dutch. This trial was registered at the Dutch Trial Register, LTR9128, www.onderzoekmetmensen.nl of which data can be found on the International Clinical Trials Registry Platform.

Participants were recruited at the obstetric outpatient clinic of the tertiary hospital Amsterdam UMC location VUmc and were included before 16 weeks of gestation. Randomisation to additional use of the mobile educative application or standard counselling alone (details are specified in Appendix A.1) was in a ratio of 1:1 and was executed by an algorithm in Castor Electronic Data Capture (EDC) clinical data management software [18]. This process was performed by the executive investigator (JW). The power calculation was to include 140 participants, 70 participants in each arm, to detect a 15% difference in aspirin adherence with 90% power and Alpha of 0.05, based on the results of Abheiden et al. [10]. To fulfil the power and taking into account a 25% loss-to-follow-up, the established population size was 175 participants.

### 2.2 Materials and outcome measures

We developed a mobile application named ‘Mijn Medicijn Zwangerschap’ (‘My Medicine Pregnancy’; available on www.adobe.ly/2yFMpt5 and the English translation of the text of the application is depicted in Appendix A.2) in collaboration with Delft University of Technology (TU Delft) [19]. This application was available in Dutch. It provides short texts using laymen's terminology and pictures on the indication of aspirin during pregnancy, benefits and risks in using the medication, risks when not using the medication, side effects and safety for the child. The duration of the reading will be estimated maximum five minutes. The application was tested previously in a pilot study (*n* = 8) at our obstetric outpatient clinic of the Amsterdam UMC location VUmc in 2019. The revised version of the application based on the input of patients in the pilot study was used in this trial. Interactive features, such as a reminder function or pill count, were not available. The use of the application was not registered.

Primary outcome of this study was aspirin adherence during pregnancy measured by two validated questionnaires and patient reported tablet intake >90%. At 26 to 30 weeks of gestation, participants received an online questionnaire which consisted of 48 questions. Participants who were allocated to the mobile application group had seven extra questions about the use of the mobile application, resulting in 55 questions. The questionnaire consisted of general questions on sociodemographic background and obstetric history, and two validated questionnaires on medication adherence: Simplified Medication Adherence Questionnaire (SMAQ) and Beliefs and Behaviour Questionnaire (BBQ) [20,21]. The SMAQ is a questionnaire measuring the level of self-reported adherence with six-items: four qualitative items and two quantitative items [21]. Participants were considered non-adherent if they gave either a positive response to one of the four qualitative questions or missed more than two tablets in the last week or month [21]. The SMAQ had a satisfactory internal consistency with a Chronbach's alpha of 0.75 [21]. Acceptable questionnaires are those with a Chronbach's alfa >0.70 [22]. The BBQ is a validated questionnaire which consists of 30 questions on adherence in three categories: beliefs, experience and behaviour [20]. Each category has antagonistic subscales: confidence and concerns, satisfaction and disappointment, and adherence and non-adherence. All subscales have a Chronbach's alfa >0.70, with the exception of the subscale ‘confidence’ with a Chronbach's alfa of 0.62 [20]. Participants were considered non-adherent if they had a low score on subscale ‘adherence’ (<19) and a high score on the subscale ‘non-adherence’ (>8). In both questionnaires minor modifications were made to focus on aspirin during pregnancy: ‘medication’ was replaced by ‘aspirin’ and ‘during pregnancy’ was added. Patient reported tablet intake was derived from the quantitative question of the SMAQ: ‘How often did you forget to take your aspirin last month?’ (<3 times (assuming 30 days a month, resulting in adherence >90%) or ≥ 3 times (≤90%)).

Secondary outcomes were experiences with use of the educative application: frequency of use, convenience of use, helpfulness in aspirin adherence, influence on confidence of using aspirin, rather using the application to gain information than the internet, and recommendation of the application to others. In addition, obstetric outcomes including complication of pregnancy with HDP and/or FGR, iatrogenic preterm birth due to HDP and/or FGR, mode of delivery, intra-uterine fetal death, gestational age of delivery, birthweight and dysmaturity (defined as birth weight < 10th percentile) were reported. Subgroup analyses were performed to investigate the influence of parity, maternal age, body mass index (BMI), educational level and previous pregnancy complicated by PE on aspirin adherence during pregnancy.

### 2.3 Statistical analyses

Data were examined using descriptive statistics and are depicted as mean with standard deviation (SD), median with interquartile range (IQR) or number with percentage as appropriate. To compare the data of the two groups, independent *t*-test for normally distributed numerical variables and Mann-Whitney *U* test for not-normally distributed numerical variables were used. Chi Square test or Fishers exact test was used for binary variables. Logistic regression was used to perform the subgroup analyses, resulting in odds ratio with 95% confidence interval. Statistical analyses were performed with SPSS version 28 (SPSS Inc., Chicago, IL). *P*-values of <0.05 were considered to be statistically significant.

## 3 Results

**Fig. 1:**
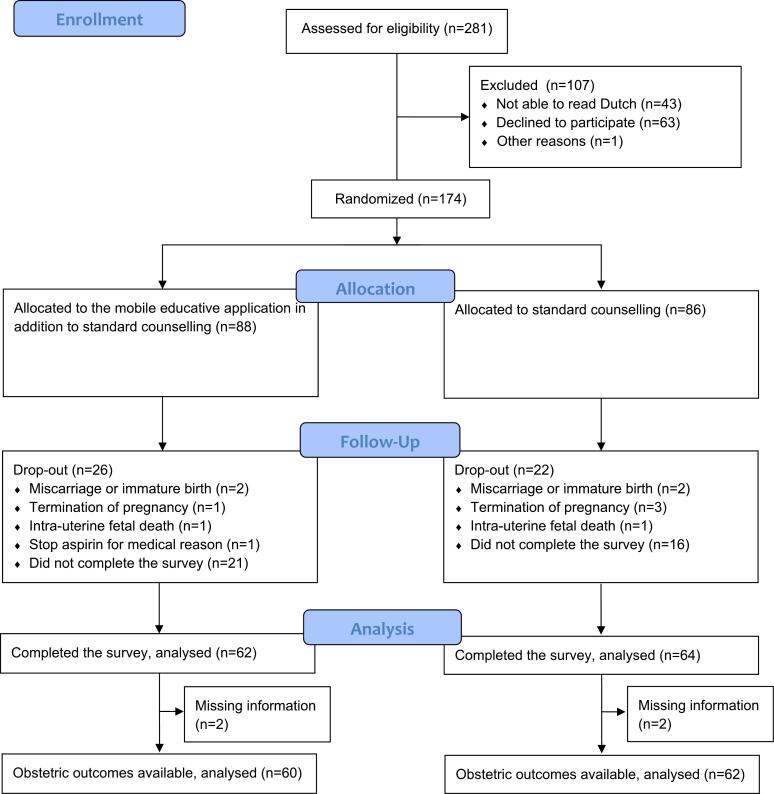
CONSORT flow diagram of participants of the ASAP study.

This randomised controlled trial was conducted from July 2020 until August 2022. In Fig. 1, the CONSORT flowchart of inclusions is displayed.

Baseline characteristics are listed in Table 1. Two statistically significant differences between the allocation groups were found: lower mean BMI and higher number of FGR in previous pregnancies in the mobile application group. The most frequently reported aspirin indication was a combination of moderate risk factors.

**Table 1:** Baseline characteristics of participants of the ASAP study.

	Mobile application (*n* = 62)	Standard counselling (n = 64)	*p*-value
Maternal age (years)	35.2 ± 4.6	35.4 ± 5.2	0.837
BMI (kg/m^2^)	23.0 ± 3.6	25.1 ± 6.1	0.004
Multiparity	21 (33.9)	17 (26.6)	0.372
Educational level*			0.198
Low	2 (3.2)	0 (0.0)	
Middle	15 (24.2)	11 (17.2)	
High	45 (72.6)	53 (82.8)	
Aspirin indication**			
Previous PE	12 (19.4)	13 (20.3)	0.893
Chronic hypertension	5 (8.1)	8 (12.5)	0.413
Diabetes type I or II	5 (8.1)	6 (9.4)	0.794
Chronic kidney disease	2 (3.2)	1 (1.6)	0.540
Autoimmune disease	11 (17.7)	10 (15.6)	0.750
Previous FGR	14 (22.6)	6 (9.4)	0.043
Moderate risk factors	16 (25.8)	19 (29.7)	0.627
Other***	8 (12.9)	13 (20.3)	0.265

Data are depicted as mean ± SD or number (%) as appropriate. BMI, body mass index; PE, preeclampsia; FGR, fetal growth restriction. * Educational level is divided in three levels based on the International Standard Classification of Education [23]. ** Summed results are >100% since women can have more than one aspirin indication. *** Other indications mobile application group (*n* = 8): preterm birth (*n* = 2), family history of cardiovascular diseases (n = 2), placental abruption (*n* = 1), Factor V Leiden mutation (n = 1), recurrent miscarriages (n = 1) and patients' preference (n = 1). Other indications standard counselling (*n* = 13): recurrent miscarriages (*n* = 4), twin pregnancy (*n* = 3), preterm birth (n = 2), placental abruption (n = 1), acute fatty liver in pregnancy (n = 1), asphyxia (n = 1) and patients' preference (n = 1).

Intention-to-treat analysis were performed. No statistically significant difference on aspirin adherence rates as measured by the SMAQ (Table 2) and BBQ (Table 3) were seen between the two groups. Tablet intake of >90% in the previous month was equal in both groups (Table 2). The results of the other subscales of the BBQ are listed in Appendix A.3.

**Table 2:** Results of the Simplified Medication Adherence Questionnaire (SMAQ). Data are depicted as number (%).

	Mobile application (n = 62)	Standard counselling (n = 64)	p-value
Qualitative questions			
Have you ever forgotten to take your aspirin? Yes	40 (64.5)	33 (51.5)	0.141
Do you always take your aspirin at the advised time? No	1 (1.6)	6 (9.4)	0.057
Did you ever stop taking your aspirin if you felt unwell/sick? Yes	8 (12.9)	7 (10.9)	0.733
Did you forget to take your aspirin last weekend? Yes	1 (1.6)	1 (1.6)	0.982

Quantitative questions			
How often did you forget to take your aspirin last week? ≥3 times	0 (0.0)	1 (1.6)	0.323
How often did you forget to take your aspirin last month? ≥3 times	7 (11.3)	8 (12.5)	0.834
Non-adherent	43 (69.4)	37 (57.8)	0.179

**Table 3:** Results of the Beliefs and Behaviour Questionnaire (BBQ), category behaviour. Mobile educative application *n* = 62, standard counselling *n* = 64. Data are depicted as number (%).

	Mobile app	Standard counsel	Mobile app	Standard counsel	Mobile app	Standard counsel	Mobile app	Standard counsel	Mobile app	Standard counsel	p-value
5-point-Likert-scale	1 = Strongly disagree		2 = Disagree		3 = Neutral		4 = Agree		5 = Strongly agree		

Behaviour Adherence											
I have strict routines for using my aspirin.	0 (0.0)	1 (1.8)	0 (0.0)	2 (3.1)	6 (9.7)	5 (7.8)	33 (53.2)	29 (45.3)	23 (37.1)	27 (42.2)	0.457
I keep my aspirin close to where I need to use them.	1 (1.6)	0 (0.6)	2 (3.2)	1 (1.6)	2 (3.2)	4 (6.3)	32 (51.6)	30 (46.9)	25 (40.3)	29 (45.3)	0.675
I ensure I have enough aspirin so that I do not run out.	1 (1.6)	0 (0.0)	1 (1.6)	1 (1.6)	5 (8.1)	2 (3.1)	25 (40.3)	33 (51.6)	30 (48.4)	28 (43.8)	0.489
I push myself to follow the instructions of my doctors.	10 (16.1)	16 (25.0)	15 (24.2)	12 (18.8)	18 (29.0)	11 (17.2)	14 (22.6)	14 (21.9)	5 (8.1)	11 (17.2)	0.229

Non-adherence											
I get confused about taking aspirin during pregnancy.	20 (32.3)	25 (39.0)	33 (51.6)	27 (42.2)	6 (9.7)	6 (9.4)	3 (4.8)	6 (9.4)	0 (0.0)	0 (0.0)	0.547
I make changes in the recommended management to suit my lifestyle.	31(50.0)	41 (64.1)	28 (45.2)	19 (29.7)	1 (1.6)	0 (0.0)	2 (3.2)	4 (6.3)	0 (0.0)	0 (0.0)	0.191
I vary my recommended management based on how I am feeling.	34 (54.8)	42 (65.6)	26 (41.9)	17 (26.6)	1 (1.6)	3(4.7)	1 (1.6)	2 (3.1)	0 (0.0)	0 (0.0)	0.258
I put up with my medical problems before taking any action.	10 (16.1)	11 (17.2)	19 (30.6)	16 (25.0)	7 (11.3)	17 (26.6)	25 (40.3)	16 (25.0)	1 (1.6)	4 (6.3)	0.084
Non-adherent	24 (38.7)	22 (34.4)									0.613

The experiences of the application are depicted in Table 4. The majority did not use the application in the month before completing the survey. More than half of the participants found the mobile-application easy to use and would recommend it to others. Obstetric outcomes are listed in Appendix A.4.

**Table 4:** Experiences with the application of the ASAP study. Data are depicted as number (%).

	n = 62
How often did you use the application in the last month?	
0 times	46 (74.2)
1 time	14 (22.6)
2–5 times	2 (3.2)
>5 times	0 (0.0)
Was the application easy to use? Yes	39 (62.9)
Did the application help you in taking your aspirin? Yes	14 (22.6)
Did the application influence your confidence in using aspirin during your pregnancy? Yes	10 (16.1)
I would rather use the application than searching for information on the internet.	

5-point-Likert scale	
- 1 = strongly disagree	4 (6.5)
- 2 = disagree	9 (15.0)
- 3 = neutral	28 (45.2)
- 4 = agree	15 (24.2)
- 5 = strongly agree	6 (9.7)
I would recommend the application to others. Yes	35 (56.5)

Subgroup analyses showed a negative effect of BMI on both aspirin adherence according to the BBQ (OR 0.927, 95%CI: 0.862–0.997; *p* = 0.043) and tablet intake >90% derived from the quantitative data of the SMAQ (OR 0.876, 95%CI: 0.800–0.960; *p* = 0.004), and a positive effect of high educational level on aspirin adherence according to the BBQ (OR 4.370, 95%CI: 1.861–10.261; *p* < 0.01).

## 4 Discussion and conclusion

### 4.1 Discussion

This RCT revealed no beneficial effect of a mobile educative application to standard counselling alone by caregivers on aspirin adherence during pregnancy. Non-adherence rates varied according to the two validated questionnaires used. Tablet intake, however, was high in both groups.

We hypothesized that a mobile educative application about aspirin during pregnancy in addition to standard counselling would improve aspirin adherence, but the results of our RCT did not confirm this hypothesis. This hypothesis was, among others, based on a systematic review in 2019 which concluded that the use of mobile apps helps in increasing therapy adherence [14]. The main difference between our study and the studies included in the review, is that our application did not sent reminders to patients for taking their medication in contrast to the studies in the review [14]. An active reminder might reduce non-intentional omission.

A rationale behind our hypothesis was that improving knowledge by offering written information would lead to better adherence. A previous study of Karunia et al. in Indonesia showed improvement of knowledge on PE and aspirin adherence after oral and written education about these subjects [15]. A group of twelve women received oral and written education one-on-one by a member of the research team. This method with intensive, personal education is in contrast with the voluntary use of a mobile educative application in our study, and might explain the different effects found.

Aspirin non-adherence rates of up to 69% and 34% on respectively the SMAQ and BBQ were found in our study. The discrepancy in adherence rates between the different methods complicates the interpretation of the results of our trial. Variation in reported adherence rates can be explained by different methods used, since consensus about the best method to measure aspirin non-adherence is lacking. In 2017, Navaratnam et al. showed that in obstetric trials, aspirin adherence was most frequently tested with semi-quantitative methods as for instance pill count, blister pack inspection and medication weighing [24]. Alternatives used were quantitative methods such as serum thromboxane B_2_ measurements or qualitative methods as for instance (validated) questionnaires, interviews or self-reporting [24]. All methods have their advantages and disadvantages and no gold standard could be advised [24]. The two validated questionnaires we used in our study can be categorized as qualitative methods. One could advocate that using quantitative methods might have led to a more reliable result, but the higher costs, more complex logistics and greater invasiveness for the participants should be also taken into account.

Highest non-adherence rates were found in the SMAQ (up to 69%). Looking into more detail to the SMAQ, even after forgetting medication once, the label of non-adherence is already given. One can imagine that in case of preventive therapy in patients without complaints non-intentional omission happens more often in comparison to patients with symptomatic diseases. Therefore, the SMAQ might not be the best method for testing aspirin adherence during pregnancy.

Secondary analyses of the ASPRE trial showed that the cut-off value of aspirin tablet intake <90% is associated with adverse outcomes [7,8]. In our trial, almost 90% of the participants had a self-reported tablet intake >90%. The high number of tablet intake in our study may be explained by social desirability bias. Also, participating in a study about aspirin adherence, may have made all participants and caregivers aware of the importance of aspirin adherence and may have resulted in good tablet intake. Thereby, the high table intake in the control group makes it difficult to achieve improvement in the intervention group.

Subgroup analyses showed a negative effect of BMI and a positive effect of educational level on aspirin adherence in our trial. In literature, several factors influencing aspirin adherence are described. Vinogradov et al. revealed the following barriers among postpartum women who were aspirin non-adherent: inadequate knowledge, and lack of identification with risk factors and being a ‘medication taker’ [25]. Shanmugalingam et al. identified similar barriers, namely pill burden and non-intention omission as negative factors, and good communication with health care providers as a positive factor [11]. Sub-analysis of the study of Vinogravod et al. showed that pregnant women and their partners seek for new interactive educational resources instead of leaflets [26].

### 4.2 Reflection

We investigated a novel, educative application on aspirin adherence in pregnancy. To our knowledge, this is the first study investigating the effect of the use of a mobile educative application on aspirin adherence during pregnancy. The close collaboration with the TU Delft in developing the mobile educative application, and testing it in the women at risk for utero-placental complications in a pilot setting, resulted in a unique, customized application which we could test in our trial. Thereby, our study was performed in a randomised setting resulting in high quality evidence. Despite these efforts, no added value of our application on aspirin adherence was found. This is in contrast with our hypothesis and earlier findings of the previous mentioned systematic review and Karunia et al. [14,15]. We will reflect on the unexpected null-finding of our RCT: lessons to be learned from our ‘brilliant failure’.

At first, our population selection turned out to be biased. The major problem that we could not reveal a difference in our trial, was the tablet intake >90%. This intake is substantially higher than the reported numbers in previous studies. If baseline tablet intake is already high, it is difficult to significantly raise those rates with an intervention. The high numbers might be explained by the fact that the great majority of the study population is highly educated (education level was positively correlated with aspirin adherence in our RCT) and that we investigated the application in a tertiary population which may receive extra care and therefore be better informed than the average pregnant population. Because of stricter privacy regulations, we were not allowed to collect data on participants' ethnicity. As a result, any effect of ethnicity could not be examined. We excluded women who were unable to read the Dutch language, since the mobile application was only available in Dutch, creating selection bias. Women with a language barrier may benefit more from access to an educative application than women without a language barrier. It is still likely that our educative application can have a beneficial effect in a lower risk population in a non-tertiary center and that an adjusted application in multiple languages may benefit non-Dutch speaking women.

Secondly, the development of the mHealth application can be improved. Digital exclusion, defined by restricted access or proficiency in digital technologies, may reduce the effect of educative applications. Awareness about this phenomenon is crucial when creating and implementing new digital resources. The pilot study performed for creating our application probably biased attracting interested and motivated women, who may not be representative for the tested population in our RCT. Thereby, we tested an educative application, solely informing the users about aspirin in pregnancy. The application did not have interactive features such as a reminder function or actively registration of pill intake. Since previous interactive applications did find a positive effect on therapy adherence, this difference might explain our null-finding. In addition, the population of previous studies differ from ours. Mostly, people with chronic illness were investigated, which is not comparable to our young, mostly healthy, pregnant population using aspirin as a preventive therapy for a short period of time. It is worth considering investigating an adjusted application including interactive elements, more tailor made to women's individual needs including medication reminders, addressing concerns, adequate health literacy and side effects, and/or offering rewards. The technology acceptance model of Davis should be used when adjusting the application [27]. This model predicts acceptance of new technology based on users experience, based on perceived usefulness and perceived ease of use resulting in an attitude towards use and eventually behavioural intention and actual use [27]. Rates of benefit of usability (62.9%) and recommendation of the application (56.5%) could be improved by using this model.

At last, study set-up impairments could play a role in our null-finding. We estimated a loss-to-follow-up of 25% and unfortunately, it was 30% and 26% in the mobile application and standard counselling group, respectively. Thus, we did not reach the calculated sample size of 70 participants per arm needed to detect a difference. According to the experiences with the application, the majority of the participants did not use the application the month prior to responding the questionnaire. Unfortunately, we did not ask how often participants used the application in the end of the first and/or beginning of the second trimester, although that period is often the window of decision making about the start of aspirin. Despite the low number of participants using the mobile application, more than 50% recommended the application to others. Thereby, testing aspirin adherence in pregnancy with validated questionnaires seemed not to be the best method, as explained previously in this discussion. We encourage researchers to investigate other methods for improving aspirin adherence during pregnancy. Thereby, consensus on which method(s) used to test aspirin adherence and what cut-off value to use must be obtained in order to compare results of different trials.

### 4.3 Conclusion

We revealed no added effect of a mobile educative application about aspirin in pregnancy to standard counselling by caregivers on aspirin adherence despite the robust set-up of the study. The theoretical background concerning the indication to enhance aspirin adherence during pregnancy and the mHealth application was promising. Our ‘brilliant failure’ was mainly caused by the equally high tablet intake in the control and study group. The lessons learned concerned bias in the high educated tertiary care study population, bias in the development of the mobile application and study set-up impairments. To obtain a heterogeneous population of patients receiving primary, secondary and tertiary care, low and high educated, and Dutch as well as non-Dutch speaking is of great importance for generalisability. Other advises are that prior to future research on this subject, consensus on counselling methods and method(s) and cut-off values to use for aspirin adherence measurements during pregnancy should be obtained. Improving aspirin counselling and adherence must be on the agenda of researchers and caregivers to improve outcomes for mother and neonate.

## Funding

This research did not receive any specific grant from funding agencies in the public, commercial, or not-for-profit sectors.

## CRediT authorship contribution statement

**Jeske M. bij de Weg:** Writing – original draft, Project administration, Formal analysis, Data curation. **Marjon A. de Boer:** Writing – review & editing, Supervision, Conceptualization. **Cynthia Meijer:** Writing – review & editing, Project administration, Formal analysis, Data curation. **Noëlle Lugtenburg:** Writing – review & editing, Resources, Conceptualization. **Marijke Melles:** Writing – review & editing, Conceptualization. **Johanna I.P. de Vries:** Writing – review & editing, Supervision, Conceptualization. **Christianne J.M. de Groot:** Writing – review & editing, Supervision, Conceptualization.

## Declaration of competing interest

The authors declare that they have no known competing financial interests or personal relationships that could have appeared to influence the work reported in this paper.

## Appendix A Supplementary data

Supplementary material 1: Detailed information on the allocation groups. Image 1

Supplementary material 2: English translation of the text of the application. Image 2

Appendix A.3. Supplementary material 3: Results of the Beliefs and Behaviour Questionnaire (BBQ), categories ‘beliefs’ and ‘experiences’. Mobile educative application n = 62, standard counselling n = 64. Image 3

Appendix A.4. Supplementary material 4: Obstetric outcomes of participants of the ASAP study. Image 4

## References

[1] Atallah A., Lecarpentier E., Goffinet F., Doret-Dion M., Gaucherand P., Tsatsaris V. (2017). Aspirin for prevention of preeclampsia. Drugs.

[2] Khan K.S., Wojdyla D., Say L., Gulmezoglu A.M., Van Look P.F. (2006). WHO analysis of causes of maternal death: a systematic review. Lancet.

[3] Magee L.A., Pels A., Helewa M., Rey E., von Dadelszen P., Canadian Hypertensive Disorders of Pregnancy Working G (2014). Diagnosis, evaluation, and management of the hypertensive disorders of pregnancy. Pregnancy Hypertens.

[4] Mol B.W.J., Roberts C.T., Thangaratinam S., Magee L.A., de Groot C.J.M., Hofmeyr G.J. (2016). Pre-eclampsia. Lancet.

[5] Bujold E., Roberge S., Lacasse Y., Bureau M., Audibert F., Marcoux S. (2010). Prevention of preeclampsia and intrauterine growth restriction with aspirin started in early pregnancy: a meta-analysis. Obstet Gynecol.

[6] Roberge S., Bujold E., Nicolaides K.H. (2018). Aspirin for the prevention of preterm and term preeclampsia: systematic review and metaanalysis. Am J Obstet Gynecol.

[7] Poon L.C., Wright D., Rolnik D.L., Syngelaki A., Delgado J.L., Tsokaki T. (2017). Aspirin for evidence-based preeclampsia prevention trial: effect of aspirin in prevention of preterm preeclampsia in subgroups of women according to their characteristics and medical and obstetrical history. Am J Obstet Gynecol.

[8] Wright D., Poon L.C., Rolnik D.L., Syngelaki A., Delgado J.L., Vojtassakova D. (2017). Aspirin for evidence-based preeclampsia prevention trial: influence of compliance on beneficial effect of aspirin in prevention of preterm preeclampsia. Am J Obstet Gynecol.

[9] Mone F., Gupta J.K., Phelan M.M., Meher S., Lian L.Y., Francis B. (2021). Platelet response to aspirin in UK and Irish pregnancy cohorts: a genome-wide approach. Platelets.

[10] Abheiden C.N., van Reuler A.V., Fuijkschot W.W., de Vries J.I., Thijs A., de Boer M.A. (2016). Aspirin adherence during high-risk pregnancies, a questionnaire study. Pregnancy Hypertens.

[11] Shanmugalingam R., Mengesha Z., Notaras S., Liamputtong P., Fulcher I., Lee G. (2020). Factors that influence adherence to aspirin therapy in the prevention of preeclampsia amongst high-risk pregnant women: a mixed method analysis. PloS One.

[12] Morawski K., Ghazinouri R., Krumme A., Lauffenburger J.C., Lu Z., Durfee E. (2018). Association of a smartphone application with medication adherence and blood pressure control: the MedISAFE-BP randomized clinical trial. JAMA Intern Med.

[13] Weisman O., Schonherz Y., Harel T., Efron M., Elazar M., Gothelf D. (2018). Testing the efficacy of a smartphone application in improving medication adherence, among children with ADHD. Isr J Psychiatry Relat Sci.

[14] Pérez-Jover V., Sala-González M., Guilabert M., Mira J.J. (2019). Mobile apps for increasing treatment adherence: systematic review. J Med Internet Res.

[15] Karunia R.I., Purnamayanti A., Prasetyadi F.O.H. (2020). Impact of educational preeclampsia prevention booklet on knowledge and adherence to low dose aspirin among pregnant women with high risk for preeclampsia. J Basic Clin Physiol Pharmacol.

[16] NVOG (2019).

[17] (2010). Hypertension in pregnancy: diagnosis and management.

[18] Castor Electronic Data Capture (2019). https://castoredc.com.

[19] Lugtenburg N. (2019).

[20] George J., Mackinnon A., Kong D.C., Stewart K. (2006). Development and validation of the Beliefs and Behaviour Questionnaire (BBQ). Patient Educ Couns.

[21] Knobel H., Alonso J., Casado J.L., Collazos J., González J., Ruiz I. (2002). Validation of a simplified medication adherence questionnaire in a large cohort of HIV-infected patients: the GEEMA study. Aids.

[22] Lance C.E., Butts M.M., Michels L.C. (2006). The sources of four commonly reported cutoff criteria - what did they really say?. Organ Res Methods.

[23] United Nations Educational, Scientific and Cultural Organization (2011).

[24] Navaratnam K., Alfirevic Z., Pirmohamed M., Alfirevic A. (2017). How important is aspirin adherence when evaluating effectiveness of low-dose aspirin?. Eur J Obstet Gynecol Reprod Biol.

[25] Vinogradov R., Smith V.J., Robson S.C., Araujo-Soares V. (2021). Aspirin non-adherence in pregnant women at risk of preeclampsia (ANA): a qualitative study. Health Psychol Behav Med.

[26] Vinogradov R., Smith V.J., Robson S.C., Araujo-Soares V. (2021). Informational needs related to aspirin prophylactic therapy amongst pregnant women at risk of preeclampsia - a qualitative study. Pregnancy Hypertens.

[27] Davis F.D. (1989). Perceived usefulness, perceived ease of use, and user acceptance of information technology. MIS Q.

