# Supplementary material for: The value of a mobile educative Application additional to Standard counselling on aspirin Adherence in Pregnancy: the ASAP study, a randomised controlled trial
Source: PEC Innov. 2024 Feb 18;4:100268. doi: 10.1016/j.pecinn.2024.100268 (PMC10907203; doi:10.1016/j.pecinn.2024.100268)
Supplement: Supplementary file 1 — Supplementary material 1: Detailed information on the allocation groups. [file mmc1.docx]

**Table A.1: Detailed information on the allocation groups**

| **Allocation to additional use of an educative mobile application** | **Allocation to standard counselling** |
| --- | --- |
| Counselling on aspirin in pregnancy by caregiver at the intake appointment. Performed <16 weeks of gestation. Counselling was done according to the Dutch national guideline (1) based on NICE Guideline (2). In approximately 5 to 10 minutes the following will be discussed: indication for use, risk reducing effect, dosing regimen, safety for mother and child and side effects. | Counselling on aspirin in pregnancy by caregiver at the intake appointment. Performed <16 weeks of gestation. Counselling was done according to the Dutch national guideline (1) based on NICE Guideline (2). In approximately 5 to 10 minutes the following will be discussed: indication for use, risk reducing effect, dosing regimen, safety for mother and child and side effects. |
| Access to an educative mobile application <16 weeks of gestation. Access was given by QR-code and url-link provided by e-mail, alongside with instructions how to use the application. The application provides short text and pictures on the indication for use, benefits and risks of aspirin, risks when not taking aspirin, side effects and safety for the child. The duration of the reading will be estimated maximum five minutes. |  |

References: 1. NVOG. NVOG Module: Acetylsalicylzuur. 2019; 2. Hypertension in pregnancy: diagnosis and management. NICE Guideline; 2010.
