# Supplementary material for: The value of a mobile educative Application additional to Standard counselling on aspirin Adherence in Pregnancy: the ASAP study, a randomised controlled trial
Source: PEC Innov. 2024 Feb 18;4:100268. doi: 10.1016/j.pecinn.2024.100268 (PMC10907203; doi:10.1016/j.pecinn.2024.100268)
Supplement: Supplementary file 2 — Supplementary material 2: English translation of the text of the application. [file mmc2.docx]

**Appendix A: English translation of the text of the application**

**My Medicine Pregnancy**

Patient-friendly information about your medication and safe use during pregnancy.

**Aspirin 80mg tablets**

Your doctor advised you this medicine because you have an increased risk of problems with the development of the placenta. As a result, you may develop preeclampsia and/or your baby may not grow well.

**What do I use this medicine for?**

Aspirin makes your platelets clot less. This lowers the risk of developing preeclampsia and/or growth retardation of the baby if you are at increased risk for these conditions.

Aspirin only works to prevent preeclampsia and/or growth retardation of your baby. It is not used to treat these conditions when they have already occurred.

**What are the risks if I don't use this medicine?**

If you do not take this medication, you are more likely to develop preeclampsia and/or growth retardation of your child because you are at increased risk.

**Why do I have an increased risk?**

You are at increased risk because you developed preeclampsia or had severely elevated blood pressure during a previous pregnancy. You also have an increased risk if you have diabetes, chronic kidney disease or a certain autoimmune disease. You may also have some smaller risk factors such as advanced age, obesity, a twin pregnancy etcetera that put you at increased risk.

**What is preeclampsia?**

Preeclampsia is a pregnancy complication characterized by a combination of hypertension and protein loss through the urine. This is dangerous for you and your unborn child:

- With preeclampsia, the placenta does not work as well, so your baby gets fewer nutrients. This can lead to growth retardation of your baby and possibly premature birth.
- In addition, your organs such as the kidneys, liver and blood vessels may not work as well.

*Read more about preeclampsia.*

**Preeclampsia***Pregnancy complication*

Preeclampsia results from a problem in the development of the placenta. This happens early in pregnancy. As a pregnant woman, you don't notice this. Later in pregnancy, you may experience preeclampsia.

Complaints that can fit with preeclampsia are headaches, problems with seeing, tight feeling in the upper abdomen, tingling sensation in your fingers and lots of fluid retention. If you experience these symptoms, we recommend you to contact your treating physician. Some women with preeclampsia have no symptoms at all.

**Is this medicine safe for my child?**

This medicine is safe for your child.

**What is known from research?**

Scientific research shows that aspirin (in doses up to 100mg per day) can be used safely during pregnancy. There is no evidence that this medicine leads to abnormalities in your baby.

In fact, aspirin helps to lower the risk of harm to your baby by reducing the risk of preeclampsia and its complications.

**Experience with the drug**

Aspirin has been used by pregnant women for many years with no adverse effects on the baby.

**What else do I need to know?**

The use of 80mg aspirin as a platelet inhibitor during pregnancy is safe. There are no adverse effects for either mother or child.

**What side effects can I get?**

Aspirin is a drug with few side effects. Most pregnant women experience no symptoms.

Side effects that sometimes occur are bruising or slower stopping of bleeding of small wounds. This is due to aspirin's inhibitory effect on platelets.

Also, stomach ache can sometimes occur. In that case, we advise you to take the medication during or after eating. If the symptoms persist, consult your doctor.

These side effects can be bothersome but are not dangerous.

**Can I take this medicine with other medications?**

If you are taking other medications besides aspirin, ask your treating physician if these medications may be taken together with aspirin.

**How do I use this medicine?**

- Take one tablet a day with a whole glass of water.
- We recommend taking the tablet in the evening for the best effect of aspirin.
- If you develop stomach ache quickly, take the tablet during or after eating.

**How do I store this medicine?**

Aspirin should be stored in sealed containers at room temperature (max. 25 degrees Celsius).

Do not use aspirin after the expiration date. You can find this on the package after "Do not use after" or "Exp".

*Do you have questions or concerns? Please discuss them with your doctor.*
