# Supplementary material for: The value of a mobile educative Application additional to Standard counselling on aspirin Adherence in Pregnancy: the ASAP study, a randomised controlled trial
Source: PEC Innov. 2024 Feb 18;4:100268. doi: 10.1016/j.pecinn.2024.100268 (PMC10907203; doi:10.1016/j.pecinn.2024.100268)
Supplement: Supplementary file 3 — Appendix A.3. Supplementary material 3: Results of the Beliefs and Behaviour Questionnaire (BBQ), categories ‘beliefs’ and ‘experiences’. Mobile educative application n = 62, standard counselling n = 64. [file mmc3.docx]

**Table A.2:** Results of the Beliefs and Behaviour Questionnaire (BBQ), categories ‘beliefs’ and ‘experiences’. Mobile educative application n = 62, standard counselling n = 64.

|  | Mobile app | Standard counsel | Mobile app | Standard counsel | Mobile app | Standard counsel | Mobile app | Standard counsel | Mobile app | Standard counsel | p-value |
| --- | --- | --- | --- | --- | --- | --- | --- | --- | --- | --- | --- |
| 5-point-Likert-scale | 1 = Strongly disagree | | 2 = Disagree | | 3 =Neutral | | 4 = Agree | | 5 = Strongly agree | |  |
| Beliefs |  | |  | |  | |  | |  | |  |
| Confidence section | | | | | | | | | | | |
| I have sufficient understanding about the risk of my pregnancy. | 1 (1.6) | 0 (0.0) | 4 (6.5) | 2 (3.1) | 6 (9.7) | 10 (15.6) | 33 (53.2) | 34 (53.1) | 18 (29.0) | 18 (28.1) | 0.618 |
| I know what to expect from taking aspirin during my pregnancy. | 0 (0.0) | 0 (0.0) | 1 (1.6) | 4 (6.3) | 13 (21.0) | 20 (31.3) | 33 (53.2) | 21 (32.8) | 15 (24.2) | 19 (29.7) | 0.094 |
| I understand the reason to take aspirin during pregnancy. | 0 (0.0) | 0 (0.0) | 0 (0.0) | 0 (0.0) | 7 (11.3) | 5 (7.8) | 32 (51.6) | 31 (48.4) | 23 (37.1) | 28 (43.8) | 0.668 |
| I am receiving the best possible management. | 0 (0.0) | 0 (0.0) | 0 (0.0) | 0 (0.0) | 9 (14.5) | 14 (21.9) | 36 (58.1) | 35 (54.7) | 17 (27.4) | 15 (23.4) | 0.550 |
| The use of aspirin during my pregnancy is a mystery for me. | 24 (38.7) | 31 (48.4) | 34 (54.8) | 24 (37.5) | 4 (6.5) | 9 (14.1) | 0 (0.0) | 0 (0.0) | 0 (0.0) | (0.0) | 0.105 |
| My aspirin is working. | 0 (0.0) | 0 (0.0) | 2 (3.2) | 3 (4.7) | 30 (48.4) | 30 (46.9) | 24 (38.7) | 24 (37.5) | 6 (9.7) | 7 (10.9) | 0.970 |
| I had a say in using aspirin during my pregnancy. | 0 (0.0) | 0 (0.0) | 8 (12.9) | 9 (14.1) | 6 (9.7) | 12 (18.8) | 35 (56.5) | 27 (42.2) | 13 (21.0) | 16 (25.0) | 0.338 |
| Using aspirin during pregnancy lowers the risk of pregnancy complications. | 0 (0.0) | 1 (1.6) | 0 (0.0) | 1 (1.6) | 12 (19.4) | 10 (15.6) | 34 (54.8) | 34 (53.1) | 16 (25.8) | 18 (28.1) | 0.687 |
| My doctor is very knowledgeable. | 0 (0.0) | 0 (0.0) | 0 (0.0) | 0 (0.0) | 8 (12.9) | 4 (6.3) | 35 (56.5) | 36 (56.2) | 19 (30.6) | 24 (37.5) | 0.387 |
| Concerns section | | | | | | | | | | | |
| It is helpful to know the experiences with aspirin of other pregnant women. | 6 (9.7) | 5 (7.8) | 18 (29.0) | 13 (20.3) | 23 (37.1) | 27 (42.2) | 15 (24.2) | 16 (25.0) | 0 (0.0) | 3 (4.7) | 0.377 |
| Natural remedies are safer than medicine. | 8 (12.9) | 11 (17.2) | 29 (46.8) | 31 (48.4) | 24 (38.7) | 21 (32.8) | 1 (1.6) | 1 (1.6) | 0 (0.0) | 0 (0.0) | 0.871 |
| My doctor has limited management options to prevent from pregnancy complications. | 15 (24.2) | 11 (17.2) | 32 (51.6) | 29 (45.3) | 11 (17.7) | 16 (25.0) | 3 (4.8) | 6 (9.4) | 1 (1.6) | 2 (3.1) | 0.559 |
| Using any medication during pregnancy involves some risk. | 1 (1.6) | 2 (3.1) | 6 (9.7) | 9 (14.1) | 22 (35.5) | 25 (39.1) | 29 (46.8) | 27 (42.2) | 4 (6.5) | 1 (1.6) | 0.564 |
| I am on too many medication. | 17 (27.4) | 24 (37.5) | 29 (46.8) | 21 (32.8) | 14 (22.6) | 15 (23.4) | 2 (3.2) | 3 (4.7) | 0 (0.0) | 1 (1.6) | 0.451 |
| Experiences | | | | | | | | | | | |
| Satisfaction section | | | | | | | | | | | |
| My doctor is compassionate. | 0 (0.0) | 0 (0.0) | 1 (1.6) | 1 (1.6) | 15 (24.2) | 16 (25.0) | 29 (46.8) | 34 (53.1) | 17 (27.4) | 13 (20.3) | 0.818 |
| I am satisfied with the information my doctor shares with me. | 0 (0.0) | 0 (0.0) | 2 (3.2) | 0 (0.0) | 8 (12.9) | 15 (23.4) | 39 (62.9) | 36 (56.3) | 13 (21.0) | 13 (20.3) | 0.239 |
| My doctor spends adequate time with me. | 0 (0.0) | 1 (1.6) | 1 (1.6) | 0 (0.0) | 9 (14.5) | 11 (17.2) | 34 (54.8) | 30 (46.9) | 18 (29.0) | 22 (34.4) | 0.589 |
| Disappointment section |  |  |  |  |  |  |  |  |  |  |  |
| I am concerned about the side effects of aspirin. | 14 (22.6) | 23 (35.9) | 39 (62.9) | 26 (40.6) | 7 (11.3) | 10 (15.6) | 2 (3.2) | 5 (7.8) | 0 (0.0) | 0 (0.0) | 0.087 |
| It is unpleasant to use aspirin during my pregnancy. | 16 (25.8) | 27 (42.2) | 34 (54.8) | 25 (39.1) | 10 (16.1) | 8 (12.5) | 2 (3.2) | 3 (4.7) | 0 (0.0) | 1 (1.6) | 0.233 |
| It is physically difficult to use aspirin. | 30 (43.4) | 43 (67.2) | 32 (51.6) | 16 (25.0) | 0 (0.0) | 1 (1.6) | 0 (0.0) | 4 (6.3) | 0 (0.0) | 0 (0.0) | 0.006 |
| Financial difficulties limit my access to the best healthcare. | 33 (53.2) | 46 (71.9) | 27 (43.5) | 15 (23.4) | 2 (3.2) | 3 (4.7) | 0 (0.0) | 0 (0.0 | 0 (0.0) | 0 (0.0) | 0.057 |
| Taking aspirin during pregnancy disrupts my life. | 32 (51.6) | 39 (60.9) | 29 (46.8) | 23 (35.9) | 1 (1.6) | 2 (3.1) | 0 (0.0) | 0 (0.0) | 0 (0.0) | 0 (0.0) | 0.431 |

Data are depicted as number (%).
