# Supplementary material for: The value of a mobile educative Application additional to Standard counselling on aspirin Adherence in Pregnancy: the ASAP study, a randomised controlled trial
Source: PEC Innov. 2024 Feb 18;4:100268. doi: 10.1016/j.pecinn.2024.100268 (PMC10907203; doi:10.1016/j.pecinn.2024.100268)
Supplement: Supplementary file 4 — Appendix A.4. Supplementary material 4: Obstetric outcomes of participants of the ASAP study. [file mmc4.docx]

**Table A.3:** Obstetric outcomes of participants of the ASAP study.

|  | **Mobile application**  **(n = 60)** | **Standard counselling**  **(n = 62)** | **p-value** |
| --- | --- | --- | --- |
| **Pregnancy complicated by HDP**  **PIH**  **PE** | 9 (15.0)  4 (44.4)  5 (55.6) | 10 (16.1)  5 (50.0)  5 (50.0) | 0.863 |
| **Pregnancy complicated by FGR** | 6 (10.0) | 5 (8.1) | 0.709 |
| **Iatrogenic preterm birth***  **<37 weeks of gestation**  **<34 weeks of gestation** | 2 (3.3)  1 (50.0)  1 (50.0) | 4 (6.5)  4 (100.0)  0 (0.0) | 0.706 |
| **Mode of delivery**  **Vaginal delivery**  **Assisted delivery**  **Caesarean section** | 35 (58.3)  3 (5.0)  22 (36.7) | 33 (53.2)  5 (8.1)  24 (38.7) | 0.763 |
| **IUFD** | 0 (0) | 1 (1.6) | 0.323 |
| **Gestational age of delivery in weeks** | 38.3 ± 1.6 | 38.0 ± 1.6 | 0.356 |
| **Birthweight in grams**** | 3112.8 ± 551.2 | 3082.2± 617.5 | 0.711 |
| **Dysmaturity**** | 9 (14.3) | 12 (17.6) | 0.600 |

Data are depicted as mean ± SD, median with [IQR] or number (%) as appropriate.
HDP, hypertensive disorders of pregnancy; PIH, pregnancy-induced hypertension; PE, preeclampsia; FGR, fetal growth restriction; IUFD, intra-uterine fetal death.
*Iatrogenic birth for the indication HDP and/or FGR. **Results based on all live born babies per group: n = 63 in the application group and n = 68 in the standard counselling group.
